# Xenopus Mcm10 is a CDK-substrate required for replication fork stability

**DOI:** 10.1080/15384101.2016.1199305

**Published:** 2016-06-21

**Authors:** Gaganmeet Singh Chadha, Agnieszka Gambus, Peter J. Gillespie, J. Julian Blow

**Affiliations:** a Centre for Gene Regulation & Expression, School of Life Sciences, University of Dundee, Dundee, UK

**Keywords:** CDK, DNA replication, Mcm10, Replication fork, Xenopus

## Abstract

During S phase, following activation of the S phase CDKs and the DBF4-dependent kinases (DDK), double hexamers of Mcm2-7 at licensed replication origins are activated to form the core replicative helicase. Mcm10 is one of several proteins that have been implicated from work in yeasts to play a role in forming a mature replisome during the initiation process. Mcm10 has also been proposed to play a role in promoting replisome stability after initiation has taken place. The role of Mcm10 is particularly unclear in metazoans, where conflicting data has been presented. Here, we investigate the role and regulation of Mcm10 in *Xenopus* egg extracts. We show that *Xenopus* Mcm10 is recruited to chromatin late in the process of replication initiation and this requires prior action of DDKs and CDKs. We also provide evidence that Mcm10 is a CDK substrate but does not need to be phosphorylated in order to associate with chromatin. We show that in extracts depleted of more than 99% of Mcm10, the bulk of DNA replication still occurs, suggesting that Mcm10 is not required for the process of replication initiation. However, in extracts depleted of Mcm10, the replication fork elongation rate is reduced. Furthermore, the absence of Mcm10 or its phosphorylation by CDK results in instability of replisome proteins on DNA, which is particularly important under conditions of replication stress.

## Introduction

The process of DNA replication is highly regulated, involving the stepwise recruitment of different factors onto replication origins in an ordered fashion. The primary purpose of this regulation is to ensure complete duplication of genetic material. From late M-phase to early G1, replication origins are licensed for subsequent activity by loading double heterohexamers of Mcm2-7. In S-phase the sequential action of Dbf4-dependent kinase (DDK) and Cyclin dependent kinase (CDK) drives the recruitment of different replication factors onto DNA-bound Mcm2-7 to generate a pre-initiation complex which includes 2 essential components, Cdc45 and the heterotetrameric GINS complex. This allows assembly of the active replicative helicase CMG (Cdc45, Mcm2-7 and GINS) which drives DNA unwinding ahead of the replication fork.

Mcm10 is an evolutionarily-conserved protein that is involved in DNA replication in different eukaryotic systems, though its exact involvement in this process is still unclear.^1^ Mcm10 was first identified in *S. cerevisiae* as a gene required for DNA replication.^2,3^ Studies in different organisms from yeast to humans have shown that Mcm10 can interact with several replication initiation factors including Mcm2-7,^2,4-10^ Cdc45,^11-13^ TopBP1^14^ and RecQ4.^15-17^

Previous studies in various organisms have implicated Mcm10 in various roles including activating the Mcm2-7 helicase in fission yeast,^18^ recruiting Cdc45 and the GINS complex to the pre-RC, stabilization of polα in yeast, *Xenopus* and humans^7,15,19-23^ and modulating chromatin dynamics in budding yeast and *Drosophila*.^24,25^ The prevalent model from *in vitro* and *in vivo* studies in budding and fission yeast is that Mcm10 plays a role late in replication initiation where it is required for unwinding of origin DNA and separation of Mcm2-7 double hexamers^10,26-29^ In addition to its involvement in DNA replication initiation, Mcm10 has also been shown to promote genomic integrity in human cells, as lack of Mcm10 leads to accumulation of DNA damage and cell cycle arrest^22,30-32^ Budding yeast Mcm10 performs some of its genome protection functions by interactions with 9-1-1 checkpoint clamp and other factors implicated in double strand break repair.^33,34^

In the current study we show that in *Xenopus* egg extracts, Mcm10 binds to chromatin at a later stage in process of DNA replication initiation in an S-CDK- and DDK-dependent manner. This is in contrast to a previous study on *Xenopus* Mcm10,^19^ but is consistent with results obtained in yeasts and other organisms. We demonstrate that *Xenopus* Mcm10 is not required for bulk DNA replication but is required for replisome stability, with depleted extracts having reduced rates of replication fork elongation. We also show that the ability of *Xenopus* Mcm10 to promote replisome stability requires it to undergo a CDK-dependent phosphorylation.

## Results

### Mcm10 chromatin binding is dependent on S-phase kinases

We raised 2 polyclonal antisera to *Xenopus* Mcm10, one against the N-terminus of the protein and one against the C-terminus. Both antibodies recognized several bands in whole egg extract, but recognized a common band at ∼100 kDa both in extract and on chromatin, as expected of *Xenopus* Mcm10 (Fig S1A). The same ∼100 kDa protein was immuno-depleted from extract by both antibodies. Mass spectrometry of immunoprecipitates from extracts and chromatin showed Mcm10 as the most abundant precipitated protein (Fig S1B).

**Figure 1.**
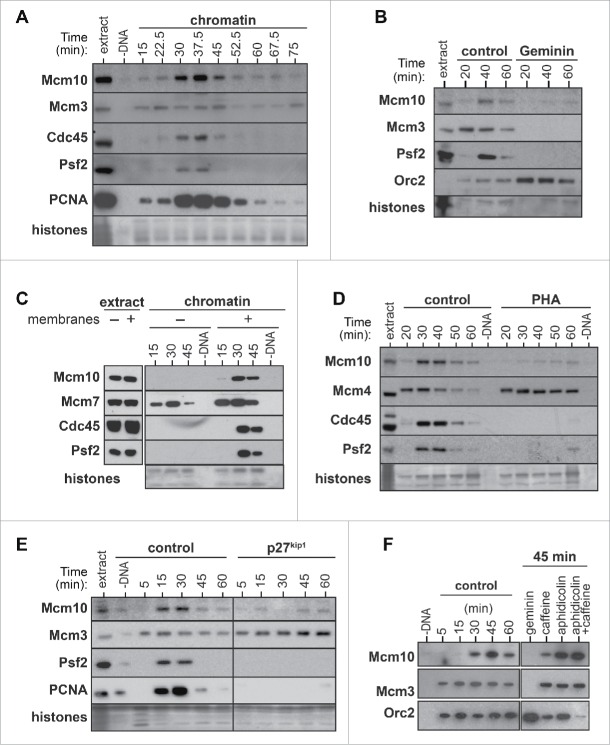
Mcm10 chromatin loading requirements. A, Xenopus egg extract was supplemented with demembranated sperm nuclei. After incubation for the indicated times, chromatin was isolated and immunoblotted for Mcm10, Mcm3, Cdc45, Psf2 and PCNA. The lower portion of the gel was stained with Coomassie to visualize histones. (B- F), Interphase (B, D, E, F) or membrane free (C) egg extracts were supplemented with demembranated sperm nuclei and were optionally supplemented with Geminin (B), 50 μM PHA-767491 (D), 100 nM p27^kip1^ (E), 40 µM aphidicolin or 5 mM Caffeine (F). At the indicated times, chromatin was isolated and immunoblotted for Mcm10, Mcm3, Mcm4, Mcm7, Orc2, Cdc45, Psf2 and PCNA.

Mcm10 chromatin binding in *Xenopus* egg extracts was previously reported to be dependent on replication licensing but independent of CDK activity.^19^ In contrast, recent reports in yeast have demonstrated that Mcm10 is loaded on chromatin at one of the last steps in the assembly of the pre-initiation complex, after both DDK- and CDK- dependent steps have been executed.^27-29^ In light of these contradictory observations in different organisms, we re-investigated the requirements for Mcm10 chromatin loading in *Xenopus* egg extracts. Consistent with its playing a role in DNA replication, *Xenopus* Mcm10 associated with chromatin precisely at the time of replication, matching the binding pattern of Cdc45, Psf2 and PCNA (Fig. 1A) which all function at active replisomes when DNA synthesis occurs (Fig S2A). As previously reported in *Xenopus* egg extract^19^ prior DNA licensing was required for Mcm10 chromatin recruitment as upon Geminin addition, Mcm10 chromatin binding was inhibited (Fig. 1B).

Once origin licensing is complete in *Xenopus* extracts, chromatin is assembled into interphase nuclei, allowing the nuclear import and activation of the DDK and CDK kinases that are required for the initiation of replication.^35-37^ When sperm DNA was incubated in extracts lacking nuclear membrane precursors, no Mcm10 chromatin recruitment was observed (Fig. 1C). Consistent with this result, when nuclei were assembled in egg extract supplemented with wheat germ agglutinin, a lectin that blocks nuclear pore formation,^38,39^ Mcm10 did not bind the DNA (Fig. S2B).

We followed up this result by determining whether Mcm10 chromatin loading in *Xenopus* extracts depends on either DDK or CDK kinase activity. When replication initiation was prevented by inhibiting DDK activity with PHA-767491^40^ or by inhibiting CDK with p27^kip1^, Mcm10 was not loaded onto chromatin (Fig. 1D, E). When replication fork elongation was inhibited with aphidicolin, levels of chromatin-bound Mcm10 remained high (Fig. 1F), but when additional dormant origin activation was induced by a combination of aphidicolin and caffeine,^41^ additional Mcm10 was loaded onto chromatin. These results show that Mcm10 loading occurs during replication initiation downstream of DDK and CDK activity, in agreement with results in *S. cerevisiae* and *S. pombe*,^27-29^ but in disagreement with the earlier study of Mcm10 in *Xenopus* egg extracts.^19^

### Mcm10 is a CDK substrate

**Figure 2.**
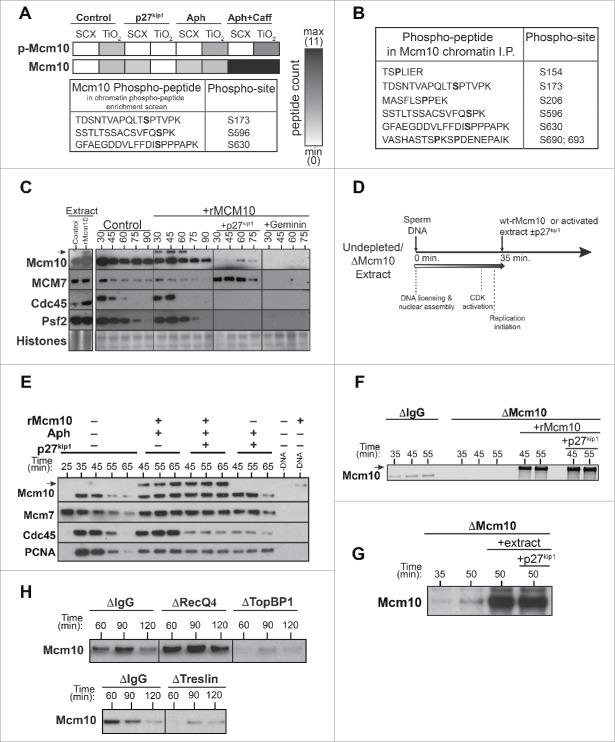
CDK-regulation of Mcm10 chromatin loading. A, B, CDK-dependent phosphopeptides of chromatin bound Mcm10 identified in mid-S-phase chromatin by phosphopeptide enrichment of total proteins (A) or direct immunoprecipitation (B) of Mcm10 followed by mass spectrometric analysis. (A) Heat map for Mcm10 phosphopeptides enrichment observed in control, CDK-inhibited (p27^kip1^), replication inhibition (aphidicolin, Aph), checkpoint-inhibition (caffeine, Caff) with black showing the presence and white the absence of a peptide. C, Extracts were supplemented with demembranated sperm nuclei with or without the addition of 1 ng/µl wt-rMcm10. Samples containing rMcm10 were optionally supplemented with p27^kip1^ or Geminin. After incubation for the indicated times, chromatin was isolated and immunoblotted for Mcm10, Mcm7, Cdc45 and Psf2. The lower portion of the gel was stained with Coomassie to visualize histones. D-G, Undepleted or Mcm10 depleted interphase egg extracts were supplemented with demembranated sperm nuclei. After incubation for 35 min, aliquots were optionally supplemented with wt-rMcm10 or undepleted interphase extract with or without p27^kip1^. At the indicated times, chromatin was isolated and immunoblotted for Mcm10 and/or Mcm7, Cdc45 and PCNA. (D) Cartoon of experimental set-up. (E) rMcm10 was added to undepleted extracts at 35 in presence of p27^kip1^, aphidicolin or both. (F, G) rMcm10 or undepleted extract was added to Mcm10 depleted extract at 35 min in presence of p27^kip1^. H, Control (nonimmune IgG), RecQ4, TopBP1 or Treslin depleted extract were supplemented with demembranated sperm nuclei. After incubation for the indicated times, chromatin was isolated and immunoblotted for Mcm10.

Previous reports have shown that *Xenopus* Cdc7 executes its essential function early in S-phase before the need for CDKs.^40,42,43^ Since Mcm10 chromatin association depends on CDK activity, we asked if Mcm10 is a direct CDK-substrate by using mass spectrometry to identify Mcm10 phosphopeptides. To determine whether any observed phosphorylation was CDK-dependent, chromatin was assembled in the presence or absence of the CDK inhibitor, p27^kip1^, and also examined the effect of aphidicolin and caffeine. Proteins were eluted from chromatin under these different conditions, digested with trypsin and bound to TiO_2_ beads or a strong cation exchanger (SCX) to enrich mono- and multiply-phosphorylated peptides, respectively. Consistent with Mcm10 chromatin loading being strictly CDK-dependent, no peptides (phosphopeptides or otherwise) were identified in chromatin isolated from p27^kip1^ samples (Fig. 2A, p27^kip1^). Mcm10 phosphopeptides were identified from all the other conditions with the highest recovery from extracts treated with aphidicolin plus caffeine, where the greatest loading of Mcm10 on chromatin was observed (Fig. 1F). Three Mcm10 phosphopeptides were identified across the different samples, each containing a phosphorylated serine followed by a proline (S173, S596 and S630, Fig. 2A), which conforms to the minimal consensus motif for CDK-phosphorylation.

In an alternative approach, Mcm10 protein was immunoprecipitated from chromatin assembled in extract that was optionally treated with p27^kip1^ and the immunoprecipitates were then analyzed by mass-spectrometry. Six different CDK-dependent phosphopeptides were identified (Fig. 2B), including the 3 identified in the general phosphopeptide enrichment screen. CDK-dependent phosphorylation of Mcm10 appears to occur only when Mcm10 is bound to chromatin as no Mcm10 phosphopeptides were observed when Mcm10 was immunoprecipitated from egg extract without DNA (data no shown).

### Mcm10 phosphorylation is not required for chromatin loading

When recombinant GST-tagged Mcm10 protein (rMcm10) expressed in *E. coli* was added to *Xenopus* extract, it was loaded onto chromatin in a manner that resembled endogenous Mcm10 (Fig. 2C). The peak of rMcm10 chromatin binding occurred during DNA replication, coinciding with the binding of Cdc45 and Psf2. Moreover no rMcm10 binding took place when licensing was inhibited with geminin or when CDK activity was inhibited with p27^kip1^. We next addressed whether CDK-phosphorylation is a pre-requisite for Mcm10 chromatin binding with experiments that are outlined in Figure 2D. In the first set of experiments, rMcm10 was added to extracts in S-phase with or without p27^kip1^; extract was also supplemented with aphidicolin at this time to prevent the termination of replication forks already active at the time of p27^kip1^ addition. Figure 2E shows that inhibiting CDK activity with p27^kip1^ did not block the chromatin loading of rMcm10. This suggests that prior phosphorylation of Mcm10 protein by CDKs is not essential for is chromatin binding.

*Xenopus* Mcm10 has the potential to self-associate via its N-terminal coiled-coil domain^44^ and human Mcm10 has also the potential to form a homo-hexameric structure.^45^ We therefore used Mcm10-depleted extracts to rule out the possibility that interactions with endogenous Mcm10 already bound to chromatin at the time of addition might allow unphosphorylated rMcm10 to bind chromatin. DNA was incubated in Mcm10-depleted extract and at 35 minutes rMcm10 was added with or without p27^kip1^ Figure 2F shows that rMcm10 was loaded onto chromatin regardless of the co-addition of p27^kip1^.

In order to rule out the possibility that an unusual feature of rMcm10 not present on the endogenous protein makes it able to bind chromatin without being phosphorylated by CDKs, we performed similar experiments with endogenous Mcm10 in place of rMcm10 (Fig. 2G). DNA was incubated for 35 min in extract immunodepleted of Mcm10, at which time the depleted extract was supplemented with an aliquot of undepleted extract with or without p27^kip1^ Figure 2G shows that endogenous Mcm10 was loaded onto chromatin regardless of the co-addition of p27^kip1^. Together, these results suggest that although the association of Mcm10 with chromatin requires CDK activity, Mcm10 itself does not need to be phosphorylated by CDKs to allow this to happen.

We next examined which of the known replication initiation factors are required for Mcm10 chromatin loading, by examining the binding of Mcm10 to chromatin in extracts depleted of TopBP1, Treslin or RecQ4. Figure 2H shows that Mcm10 chromatin loading requires TopBP1 and Treslin, but does not require RecQ4. Given that in *Xenopus* egg extracts RecQ4 is required late in the initiation process, after Cdc45 and GINS recruitment,^46-48^ this suggests that Mcm10 is also loaded onto chromatin at this late stage.

### Mcm10 is required for replication fork elongation and stability

**Figure 3.**
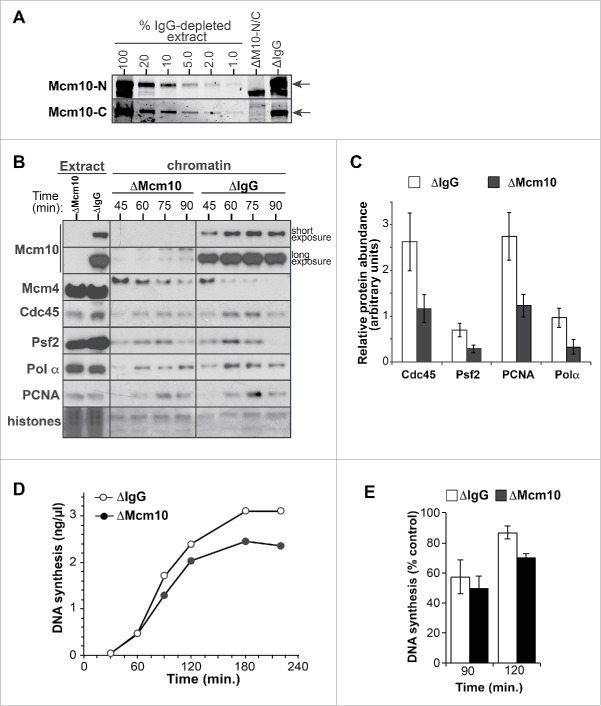
Lack of Mcm10 leads to reduction in chromatin bound replisome proteins. A, Egg extract was immunodepleted with either nonimmune IgG or Mcm10 antibodies (raised against N- or C-terminus of the protein). So that the efficiency of depletion could be estimated, 0.5 µl of each of the depleted extracts and known amounts of nonimmune-depleted extract was immunoblotted for Mcm10 using 2 different antibodies. B, Control (nonimmune IgG) and Mcm10 depleted extract were supplemented with demembranated sperm nuclei. After incubation for the indicated times, chromatin was isolated and immunoblotted for Mcm10, Mcm4, Cdc45, Psf2, Pol α and PCNA. The lower portion of the gel was stained with Coomassie to visualize histones. C, Quantitation of Cdc45, Pol α, PCNA and Psf2 bound to chromatin at 60 min in control and Mcm10 depleted extracts. Mean and SEM of 3 independent experiments is shown. D, E, Control and Mcm10 depleted extracts were supplemented with 3 ng DNA/µl and [α-^32^ P]dATP; total DNA synthesis was determined at the indicated times. (E) Mean incorporation of [α-^32^ P]dATP at 90 and 120 min. and SEM of 3 independent experiments is shown.

To understand the function of Mcm10 in DNA replication, we removed >99% Mcm10 from interphase *Xenopus* egg extracts by immunodepletion using antibodies raised against the N- or C-terminus of the protein individually (Supp. Fig S1A) or when mixed together (Fig. 3A). Mcm10 immunodepletion did not prevent nuclear assembly (data not shown). No Mcm10 could be observed on chromatin in the immunodepleted extracts (Fig. 3B and Supp. Fig S1A). Unlike the previous report on *Xenopus* Mcm10,^19^ extracts depleted of Mcm10 with our 2 antibodies (used together) still loaded Cdc45, GINS, Polα and PCNA onto DNA (Fig. 3B). However the quantity of these proteins bound to chromatin was reproducibly reduced to ∼50% of control levels in Mcm10-depleted extracts (Fig. 3B, 3C and Supp. Fig S3C). Similarly, although Mcm10-depleted extracts could still replicate DNA, they did so at a slower rate and less efficiently than control-depleted extracts (Fig. 3D, 3E and Supp. Fig S3A and S3B). Since Mcm10-depleted extract still managed to replicate the bulk of input DNA, this suggests that Mcm10 is either not required for initiation in *Xenopus* extracts or performs a function that can be taken over by another protein. A modest rescue in the amount of replication was observed upon addition of wt-Mcm10 to Mcm10 depleted extract (Supp. Fig S3D). However, the consistently slower replication rate and reduction in replication factors on chromatin in the absence of Mcm10 suggests that there could be problems with the elongation or stability of replisomes.

**Figure 4.**
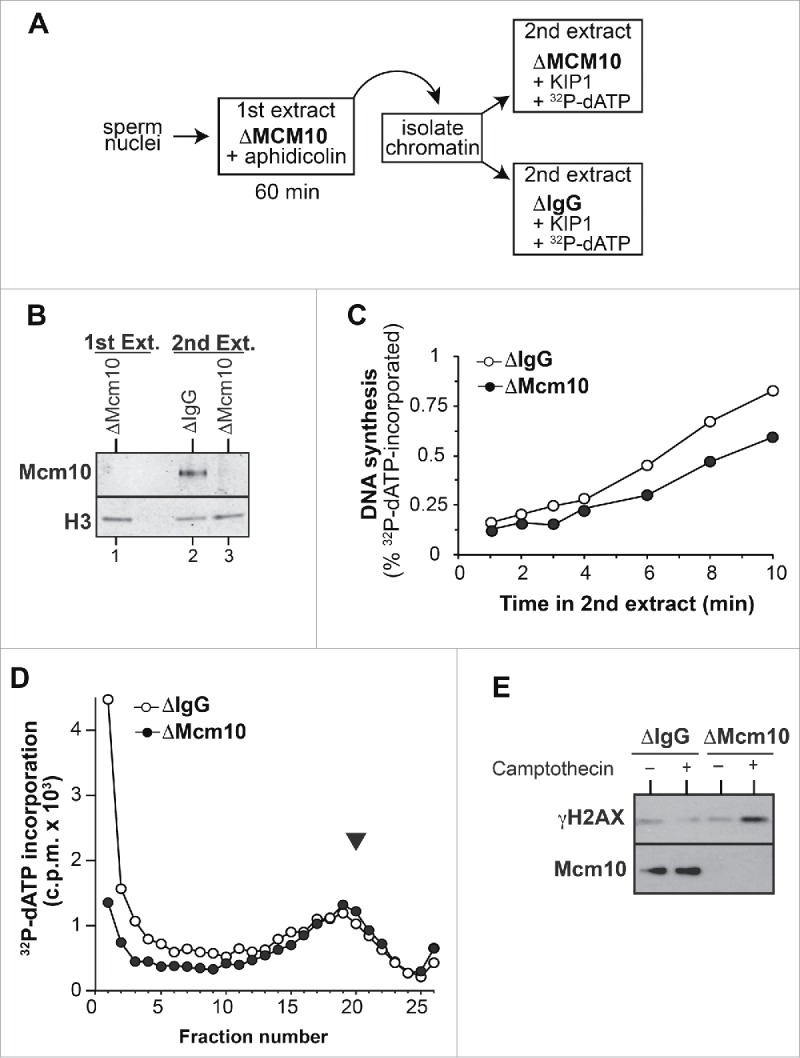
Mcm10 loss affects replication fork elongation and replisome stability. A-C, Mcm10-depleted interphase egg extracts were supplemented with demembranated sperm nuclei and 100 µM aphidicolin. After 60 min incubation, chromatin was isolated. Chromatin was then incubated in non-immune or Mcm10 depleted extract supplemented with 100 nM p27^kip1^ and optionally with [α-^32^ P]dATP. (A) Cartoon of experimental set-up. (B) Chromatin isolated after incubation in the first (lane 1) and second extracts (lane 2, 3) was immunoblotted for Mcm10 and histone H3. (C) At the indicated times, incorporation of [α-^32^ P]dATP into nascent DNA strand was determined. (D) Control (nonimmune IgG) and Mcm10 depleted extract were supplemented with 15 ng DNA/µl. At 120 mins [α-^32^ P]dATP was added for 15 seconds, and then DNA was isolated and separated on alkaline sucrose gradients. The ^32^ P content of fractions was determined by scintillation counting. The black arrowhead shows the migration of tRNA in a parallel neutral sucrose gradient. (E) Non-immune and Mcm10 depleted extract were supplemented with demembranated sperm nuclei and optionally supplemented with 50 µM camptothecin. At 60 min., chromatin was isolated and immunoblotted for Mcm10 and γH2A-X.

We directly addressed whether Mcm10 is required for fork elongation with the experiment outlined in Figure 4A.^40,49-51^ Sperm nuclei were incubated in Mcm10-depleted extract in the presence of 100 µM aphidicolin, so that forks could initiate but were unable to move away from replication origins. After 60 min, chromatin lacking bound-Mcm10 was isolated and transferred to either non-immune depleted or Mcm10-depleted extract containing p27^kip1^ to prevent any further initiation events from occurring. Replication fork progression rate was measured as the [α-^32^P]dATP incorporated over next 10 minutes. Forks in Mcm10 depleted extract, lacking chromatin bound Mcm10 (Fig. 4B), progressed at a slower rate than in non-immune-depleted extract (Fig. 4C). Measuring the overall rate of replication in 8 independent experiments gave a mean reduction in the replication rate of 0.81±0.05 (mean ± s.e.m.) in Mcm10-depleted extract compared to the non-immune-depleted controls. This suggests that the reduced rate of bulk DNA replication observed in Mcm10 depleted extract results from a reduced rate of fork elongation.

In order to examine the effect of Mcm10 depletion we pulse-labeled nascent DNA very briefly (15 seconds) with ^32^P-dATP and then separated nascent DNA on a denaturing alkaline sucrose gradient. This shows ^32^P-dATP incorporation into both high molecular weight and Okazaki-fragment-sized DNA; if the ^32^P-dATP is chased with unlabelled dATP, the Okazaki fragment-sized DNA is rapidly ligated into high molecular weight DNA, demonstrating semi-discontinuous DNA synthesis in *Xenopus* egg extracts.^52^ After a 15 second pulse of ^32^P-dATP in extract immunodepleted of Mcm10, there was a relative lack of label in high molecular weight DNA (Fig. 4D). This suggests that a lack of Mcm10 causes abnormal processing of nascent DNA at the replication fork, either due to defects in leading strand synthesis or in the ligation of Okazaki fragments.

In order to determine effect of Mcm10 on the stability of replication forks, we challenged DNA replication by incubating sperm chromatin in control and Mcm10-depleted extracts supplemented with 50 µM camptothecin, a topoisomerase I inhibitor. Camptothecin stabilizes complexes consisting of topoisomerase-I covalently linked to DNA, thereby providing an impediment to replication forks which can result in double strand DNA breaks and checkpoint activation. When DNA was replicated in Mcm10-depleted extracts there was a very large increase in γH2AX on the chromatin, suggesting the formation of a large number of double strand DNA breaks (Fig. 4E).

**Figure 5.**
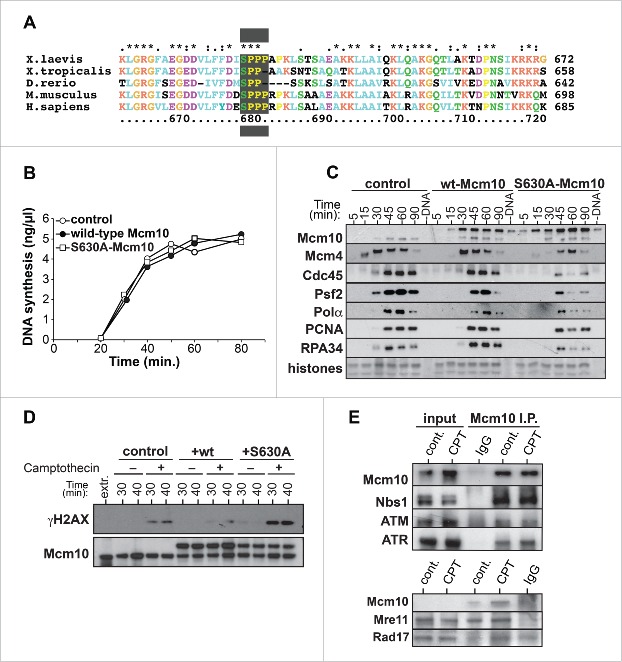
CDK-dependent Mcm10 phosphorylation is important for its function. A, Comparison of a segment of Mcm10 protein sequence alignment from *Xenopus laevis, Xenopus tropicalis, Dana rerio, Mus musculus* and *Homo sapiens*. B, C, Interphase extract was supplemented with demembranated sperm nuclei and optionally supplemented with [α-^32^ P]dATP, wt-Mcm10 or S630A-Mcm10. (B) At the indicated times, DNA synthesis was determined by measuring [α-^32^ P]dATP incorporation. A representative of 3 independent experiment is shown. (C) Chromatin was isolated at indicated times and immunoblotted for Mcm10, Mcm7, Cdc45, Psf2, Polα, or PCNA. The lower portion of the gel was stained with Coomassie to visualize histones. (D) Interphase extract was supplemented with demembranated sperm nuclei and optionally supplemented with wt-Mcm10, S630A-Mcm10 or 50 µM camptothecin. After incubation for the indicated times, chromatin was isolated and immunoblotted for Mcm10 and γH2A-X. (E) Interphase extract was supplemented with demembranated sperm nuclei and optionally supplemented with 50 µM camptothecin. At 45 min. chromatin Mcm10 was immunoprecipitated using antibodies against Mcm10 and samples were immunoblotted for Mcm10, ATR, ATM, Nbs1, Mre11 and Rad17.

Figure 2 shows that Mcm10 is phosphorylated by CDKs, but this is not essential for its chromatin loading. To investigate a potential function for Mcm10 phosphorylation by CDKs we aligned the Mcm10 protein sequence from various vertebrates. Interestingly among the different phosphorylation sites we identified on *Xenopus* Mcm10 (Fig. 2A, B) only S630 was well conserved among *Xenopus*, zebrafish, mouse and human (Fig. 5A and data not shown). We therefore synthesized recombinant Mcm10 with serine 630 replaced by alanine (Mcm10 S630A). No major effect on bulk DNA synthesis was observed when wild-type or S630A rMcm10 was added to *Xenopus* extract (Fig. 5B). However, addition of recombinant S630A Mcm10 to extracts caused a marked reduction in the abundance of CMG components (Cdc45 and Psf2) and DNA polymerase-α on chromatin (Fig. 5C). This reduction was similar in magnitude to the effect of depleting Mcm10 from extract (Fig. 3).

We therefore asked whether S630A-Mcm10 caused the loss of replisome proteins from replication forks when challenged by replication stress. Replication forks were allowed to initiate in *Xenopus* extracts in the presence of camptothecin and rMcm10 proteins. Figure 5D shows that addition of S630A-Mcm10 led to an increase in the amount of γH2AX on DNA that was induced by camptothecin. Remarkably, addition of wt-Mcm10 reduced the amount of γH2AX relative to the amount generated in untreated extract.

These results show that although Mcm10 is not essential for bulk DNA replication in *Xenopus* egg extracts, it plays an important role in protecting replisome stability and preventing the generation of double strand DNA breaks during replication. This is consistent with work in other organisms which have implicated Mcm10 in preventing DNA damage during S phase and stabilizing replisomes.^20,22,30,33,53,54^ We therefore examined whether we could detect interactions between Mcm10 and DNA repair factors at active replication forks in the *Xenopus* system. In immunoprecipitates of Mcm10 from replicating chromatin we identified interactions with the different subunits of MRN complex (Mre11, Rad17 and Nbs1) as ATM and ATR (Fig. 5E). Treatment with camptothecin did not result in further increase in interaction between Mcm10 and MRN proteins. These results are consistent with Mcm10 working with the MRN complex and checkpoint kinases to respond to problems occurring during replisome progression that could lead to double strand break formation.^34^

### Discussion

Mcm10 is one of several replication factors that form the replisome and it plays a role in ensuring the accurate replication of the genome. Despite intensive study, the precise role of Mcm10 in DNA replication still remains somewhat mysterious, with apparently conflicting results in different model systems.^1^ In the current study we have used *Xenopus* egg extracts to demonstrate that Mcm10 chromatin binding is a late event in the process of replication initiation and requires prior action of DDK and CDK. We also show that Mcm10 is a CDK substrate that requires binding of other CDK-dependent replisome factors for its chromatin association. We show that lack of Mcm10 or its phosphorylation by CDK results in instability of replication fork proteins on DNA, which is crucial particularly under conditions of replication stress.

### Mcm10 chromatin association

Previous work in *S. cerevisiae* and *S. pombe* has shown that the CMG complex assembles in the absence of Mcm10,^20,26-29^ and that Mcm10 loading occurs only after CMG assembly, thus post DDK and CDK activities.^55^ However, a previous study of DNA replication in *Xenopus* egg extracts showed Mcm10 loading to be independent of CDK or DDK activities and to be required for Cdc45 loading.^19^ We therefore revisited the kinetics and timing of Mcm10 chromatin association in *Xenopus* cell free extracts. We showed that Mcm10 loading onto chromatin depends on origin licensing, nuclear assembly and both DDK and CDK activities. We also observed increased Mcm10 loading upon hyper-activation of dormant origins by simultaneous treatment with both aphidicolin and caffeine.^41^ These results show that while Mcm10 chromatin binding occurs at the time of replication initiation, it is downstream of DDK and CDK activities. CDK- and DDK-dependent association of Mcm10 with replication origins has recently been reported in human cells,^56^ and we conclude that this is likely to be a conserved feature of Mcm10 regulation in eukaryotes.

### Requirement for Mcm10 in replication initiation

Mcm10 has been implicated in several replication-related functions, but the prevailing model in budding yeast suggests that the essential function of Mcm10 is at the step of DNA unwinding to allow RPA loading.^26-29^ However, we show that *Xenopus* extracts depleted of Mcm10 can load RPA and support bulk DNA replication at an efficiency of ∼80% of that of control extracts. Although impossible to completely rule out, we think it is very unlikely that this DNA replication depends on trace amounts of Mcm10 remaining in the depleted extracts. Immunodepletion removed >99% of total Mcm10 from the extract, and no Mcm10 was detectable on chromatin assembled in the depleted extract. Our results are consistent with studies in other metazoan cell types, where knockdown of Mcm10 by RNAi in *C. elegans*,^57^ Drosophila KC cells^11^ and Hela cells^30^ resulted in no major block to bulk DNA replication. More dramatically, *Drosophila* Mcm10 mutants were not defective for adult cell proliferation, though they were essential for embryo viability and tissue types, like compound eye, for replication and differentiation.^58^

### CDK phosphorylation of Mcm10

We have provided evidence that Mcm10 is a direct substrate of S-phase CDKs. We show that CDK phosphorylation of Mcm10 is not a pre-requisite for its loading onto chromatin but only occurs once Mcm10 is on chromatin. This is in apparent contrast to the human Mcm10 protein, which is phosphorylated at the onset of metaphase to promote its release from the chromatin.^5^ While investigating CDK regulation of Mcm10 we identified, among number of phosphorylations sites, S630 as crucial for Mcm10 function. The potential importance of S630 is indicated by its conservation in vertebrates through *Xenopus* to humans. When added to extract, a non-phosphorylatable S630A mutant of Mcm10 resulted in a phenotype that showed similarities with Mcm10 depletion: reduced recruitment of replisome proteins (Cdc45, Psf2, PCNA, polα) and a strongly enhanced γ-H2AX signal when extracts were challenged with camptothecin.

### Mcm10 and replisome stability

Although Mcm10-depleted *Xenopus* extract supported bulk DNA replication, we observed that replication was unusual in several ways. In Mcm10-depleted extracts, the replication was slightly slower and less extensive than normal, the rate of replication fork elongation was reduced, the chromatin recruitment of replisome proteins (Cdc45, Psf2, PCNA, polα) was reduced and there was a strongly enhanced chromatin recruitment of γ-H2AX when extracts were further challenged with camptothecin. Similar defects were seen in the non-phosphorylatable S360A mutant of Mcm10. These results are also consistent with the effect of Mcm10 knockdown in Drosophila KC cells^11^ and Hela cells^22,30,31^ where various features of aberrant replication were detected.

**Figure 6.**
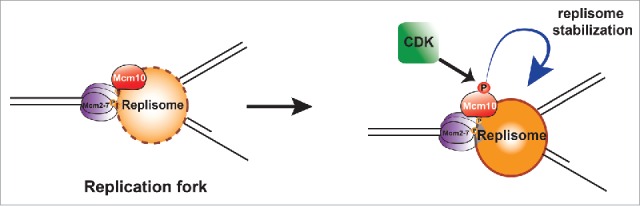
The role and regulation of Mcm10 in *Xenopus* egg extracts. Replication forks are shown, with DNA in black, the CMG helicase in purple, Mcm10 in red, and other replisome proteins in orange. Mcm10 is required for stability of the other replisome proteins, and this effect is enhanced when Mcm10 is phosphorylated by CDK.

The ∼20% reduction in fork elongation rate in *Xenopus* extracts lacking Mcm10 is similar to the reduction in replication of bulk DNA in the extracts, consistent with the idea that the primary defect is in replisome progression. Since Mcm10 has been shown to interact with multiple replisome proteins^4-17^ and is a component of moving replisomes,^59^ the slowed fork elongation rate in Mcm10-depleted extracts could likely result from a reduced stability of replisome proteins at the fork. This is consistent with the significant reduction in chromatin-bound replisome proteins that we observed in Mcm10-depleted extracts. The fact that the large reduction in replisome proteins was associated with only a modest reduction in replication rate could be due to the presence of a large number of licensed but dormant origins which can be activated if replication forks stall.^41,60^ Taken together, these observations are consistent with the primary role of Mcm10 in metazoans being to maintain or stabilizing replisomes once they have initiated (Fig. 6).

Mcm10 has been shown to interact with DNA damage repair proteins which could also explain its role in stabilizing replisomes.^1,33,61^ Alver et al.^33^ have shown that N-terminus of Mcm10 plays an important role in resisting the DNA damage induced by different genomic insults including treatment with camptothecin in budding yeast. We did not find any Mcm10 interaction with 9-1-1 complex but instead showed that *Xenopus* Mcm10 interacts with the different subunits of the MRN complex (Mre11, Rad17 and Nbs1) as well as ATM and ATR. We therefore conclude that although Mcm10 is not an essential factor for DNA replication initiation in *Xenopus*, it is an S-CDK substrate required for stabilizing other protein components at the replication fork.

## Materials and methods

### Xenopus egg extract and DNA templates

Metaphase-arrested *Xenopus laevis* egg extract and demembranated *Xenopus* sperm nuclei were prepared as described.^62^ Extracts were supplemented with 250 μg/ml cycloheximide, 25 mM phosphocreatine and 15 μg/ml creatine phosphokinase and incubated with 0.3 mM CaCl_2_ for 15 minutes to release from metaphase arrest. For DNA synthesis reactions, sperm nuclei were incubated at 6–10 ng DNA/μl in extract. DNA synthesis was assayed by measuring incorporation of [α-^32^ P]dATP into acid-insoluble material followed by scintillation counting, as described.^62^ All incubations were carried out at 23°C.

### Chromatin isolation from egg extract

Chromatin isolation for immunoblotting was carried out as described.^62^ Briefly, extract was diluted with ice-cold NIB (50 mM KCl, 50 mM mM HEPES-KOH pH 7.6, 5 mM MgCl_2_, 0.5 mM spermidine, 0.15 mM spermine, 2 mM DTT) containing phosphatase inhibitors, under-laid with NIB + 20% sucrose (w/v) and centrifuged in a swinging bucket rotor at 2100 g, 5 min, 4°C. Following a cushion wash, chromatin was compacted by spinning at 13000 g, 2 min in a fixed angle rotor. The resulting pellet was resuspended in SDS loading buffer.

Isolation of intact nuclei for transfer experiments for fork-elongation assay was carried out as described.^40^ Extracts were diluted as before but in NIB buffer where Triton X-100 was omitted, and were then under-laid with a double cushion of NIB + 20% sucrose and NIB + 30% glycerol (v:v in NIB) and centrifuged in a swinging bucket rotor. Following a cushion wash, nuclei were resuspended in the glycerol cushion and added to the second extract at a final concentration of 10 ng DNA/µl.

### Immunoblotting

For immunoblotting, samples were separated on 4–12% Bis-Tris gradient gels (Invitrogen). Proteins were transferred onto PVDF membranes (GE Healthcare, RPN303F) using a wet transfer system, blocked in PBS with 0.2% Tween-20 and 5% non-fat milk. After incubation with primary and secondary antibodies, membranes were developed using enhanced chemiluminescence detection (SuperSignal® West Pico Chemiluminescent; Thermo Scientific, 34087) or signals were acquired using Li-COR Odyssey bio-systems where Li-Cor secondary antibodies were used. The lower portion of each gel was typically cut and treated with Coomassie stain to visualize histones. Band intensities were quantified using GelEval (FrogDance Software).

### Immunoprecipitation and immunodepletion

Chromatin-bound Mcm10 from *Xenopus* egg extract was isolated as described.^62,63^ Briefly, chromatin was isolated in the middle of S-phase and resuspended in NIB+20% sucrose. To release proteins DNA was digested with 2 units/µl Benzonase (Novagen) for 10min. Samples were sonicated (Bioruptor, Diagenode) for 5 min (10 s sonication, 45 s break at medium intensity), and centrifuged 20,000 g, 4°C. The supernatant was used as an input for immunoprecipitation with Mcm10 or preimmune sheep IgG (Sigma) antibody previously bound to protein G-Dynabeads (Invitrogen) for 1 h at 4°C. For immunoprecipitation of Mcm10 in *Xenopus* egg extracts (not supplemented with DNA), interphase extracts were diluted (5-fold) in LFB 1/50 (40 mM HEPES-KOH pH 8.0, 20 mM K_2_HPO_4_/KH_2_PO_4_ pH 8.0, 2 mM MgCl_2_, 1 mM EGTA, 2 mM dithiothreitol, 10% (w/v) sucrose, 1 μg/ml each of leupeptin, pepstatin and aprotinin; supplemented with 50 mM KCl) and incubated with antibody conjugated beads as described above. The beads were then washed (5x) with filtered PBS before resuspension in loading buffer.

Immunodepletion of Mcm10 from *Xenopus* egg extract was performed as described.^62^ Briefly, metaphase arrested extract was activated to bring it into interphase. Subsequently three successive rounds of incubations of 1 volume extract with 0.2 volumes of protein G-Dynabeads beads conjugated to anti-Mcm10 antibody for 30 minutes at 4°C were carried out.

### Phosphopeptide enrichment and mass spectrometry

To enrich chromatin bound phosphoproteins, chromatin was isolated in middle of S-phase and resuspended in NIB+20% sucrose.^63^ To release proteins, DNA was digested with 2 units/µl Benzonase (Novagen) for 10min. Samples were sonicated (Bioruptor, Diagenode) for 5 min (10 s sonication, 45 s break at medium intensity), centrifuged 20,000 g, 4°C. Supernatant proteins were reduced upon incubation with 10 mM DTT at 50°C for 15 min followed by 15 min incubation with 20 mM iodoacetamide in the dark. Proteins were digested overnight with trypsin (1:100 w/w enzyme to substrate ratio) at 37°C. Samples were cleaned over ZipTip (C18 material) before sequential incubation with strong cation exchange resin (SCX) and TiO_2_ beads to enrich multiply or mono phosphorylated peptides. The extracted peptide solutions were analyzed using nano LC-MS/MS on an LTQ Orbitrap Velos (ThermoFisher, San Jose, CA).

### Recombinant proteins, reagents and antibodies

Geminin was produced as previously described.^64^ PHA-767491 and full-length p27^kip1^ were procured and used as described.^40^ Full length *Xenopus* Mcm10 was expressed from a pGEX-Mcm10 plasmid^19^ (a gift of J. Walter, Harvard Medical School) and purified from Rosetta(DE3)pLysS cells (Novagen) using glutathione–Sepharose. The purified protein was dialysed against LFB1/50 buffer. To determine protein concentration, different amounts of protein alongside known amounts of BSA were resolved by SDS-PAGE and band intensities were quantitated. Mcm10 serine to alanine point mutants were prepared from pGEX-Mcm10 using QuickChange Site-Directed Mutagenesis kit from Agilent Technologies; protein was expressed and purified as described above. Antibody against PCNA was from Santa Cruz Biotechnology. Mcm3, Mcm4, Mcm7 and Orc2 antibodies were as previously described.^65^ Cdc45, Psf2 and Polα antibodies were as previously described.^63^ RPA34 antibody was a gift from Marcel Mechali (Institute of Human Genetics, CNRS, Montpellier, France).^66^ The Mcm10 antibody was raised in sheep against a bacterially-expressed immunogen consisting of the previously described His6-tagged C-terminal (278–860 aa)^63^ and His6-tagged N-terminal (1–445 aa) fragments of *Xenopus* Mcm10 expressed and purified from *Escherichia coli* (RosettaTM (DE3)pLysS, Novagen) using Ni^2+^-NTA affinity chromatography. Mcm10-C antibody was used for Western blotting unless stated otherwise. The antiserum was affinity purified prior to use (see Fig. S1). Antibodies were used for immunoblotting at 1:1000 dilutions. Sheep polyclonal antisera were raised against synthetic peptides corresponding to sequences of ATR (MATDPGLEMASMIPALREC), Nbs1 (CEDLFRYNPKPSKRRR), Rad17 (KIEEYDSDCKIEEYDSD), ATM (DEVDLNATLGGDDPE) and Mre11 (MSSSSSSLDDEDTFKC). Immunization and antibody purification were performed as described.^67^ Blots of these antibodies against whole *Xenopus* egg extract or replicating chromatin isolated from egg extract is shown in Supplementary Figure S4.

### Alkaline sucrose gradients

Alkaline sucrose gradients were performed as described.^52^ Sperm nuclei were incubated for 120 min at 15 ng DNA/µl in 80 µl of either non-immune-depleted or Mcm10-depleted extract. The extract was then supplemented with 5 μl [α-^32^ P]dATP (10 mCi/ml). The pulse was stopped by the addition of 1 ml Buffer A (60 mM KCl; 15 mM Tris-HCI, pH 7.4; 15 mM NaCI,;1 mM β-mercaptoethanol; 0.5 mM spermidine: and 0.15 mM spermine) at 0°C. Nuclei were centrifuged at 2500 x g for 5 min, and then resuspended in 190 μl 0.5% SDS, 20 mM Tris- HCI (pH 7.4), and 20 mM EDTA. The DNA was denatured at 20°C by the addition of 10 μl 10 M NaOH. Samples were layered on top of a 5-20% sucrose gradient in 0.5 M NaCl, 0.25 M NaOH, 1 mM EDTA, and centrifuged at 55,000 rpm in an SW60Ti rotor at 20°C for 5 hr. Fractions were collected onto Whatman GF/C glass fiber filters previously soaked in saturated Na_4_P_2_0_7_, and 10 μg/ml calf thymus DNA. After drying, filters were TCA precipitated, washed in ethanol, dried again, and subjected to scintillation counting.

## Supplementary Material

Supplemental Files
