# Supplementary material for: Xenopus Mcm10 is a CDK-substrate required for replication fork stability
Source: Cell Cycle. 2016 Jun 21;15(16):2183–95. doi: 10.1080/15384101.2016.1199305 (PMC4993430; doi:10.1080/15384101.2016.1199305)
Supplement: Supplemental Files [file kccy-15-16-1199305-s001.pdf]

A

Western Blot: **Mcm10-N** Western Blot: **Mcm10-C**

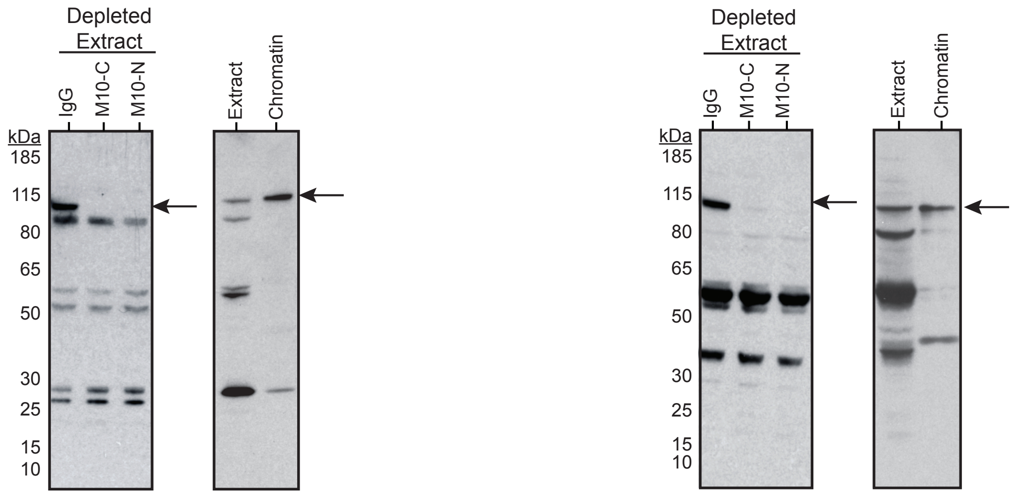

B

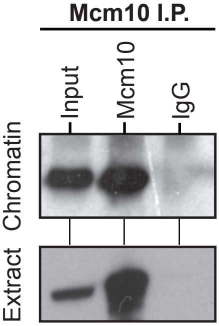

Mass spectral analysis:

Mcm10 Chromatin I.P.

Protein Description	Peptide Count	
	Mcm10	IgG
1. Mcm10p [Xenopus laevis]	35	0
2. Minichromosome maintenance protein 2; xMCM2	34	18
3. HIRA protein [Xenopus laevis]	28	4
4. Origin recognition complex protein 1 [Xenopus laevis]; XORC1 protein	22	3
5. Minichromosome maintenance protein 4-B; xMCM4-B	19	4
6. Egg envelope component ZPAX [Xenopus laevis]	17	6
7. LOC100049148 protein [Xenopus laevis]	6	0
8. MGC83928 protein [Xenopus laevis];Coiled-coil domain-containing protein 52	5	0
9. Actinin, alpha 4 [Xenopus laevis]	2	0

Mcm10 Extract I.P.

Protein Description	Peptide Count	
	Mcm10	IgG
1. Mcm10p [Xenopus laevis]	13	0
2. DNA endonuclease RBBP8 [Xenopus laevis]	9	0
3. HIRA protein [Xenopus laevis]	6	0
4. Actinin, alpha 4 [Xenopus laevis]	6	0
5. Importin beta 1 [Xenopus laevis]	5	3
6. Nucleoporin 107kDa [Xenopus laevis]	4	0
7. MGC83928 protein [Xenopus laevis]; Coiled-coil domain-containing protein 52	4	0
8. DEAD (Asp-Glu-Ala-Asp) box polypeptide 42 [Xenopus laevis]	2	0
9. Gelsolin [Xenopus laevis]	2	0

Supplementary Figure S1. Characterisation of Mcm10 antibodies in *Xenopus*.
A, 0.5 µl *Xenopus* egg extract, chromatin isolated from early S phase (40 minutes) and 0.5 µl extract depleted using antibodies against N-terminus (M10-N) or C-terminus (M10-C) or pre-immune IgGs were separated by SDS-PAGE and immunoblotted with the *Xenopus* Mcm10-N antibody (right panel) and *Xenopus* Mcm10-C antibody (leftpanel). **B**, Immunoprecipitations (IP) from *Xenopus* egg extract or from chromatin isolated in mddle of S-phase using Mcm10 (C) antibody or control sheep IgG (Sigma) previously coupled to Protein-G Dynabeads. IP samples were run on a 4–12% gradient NuPAGE gel (Invitrogen). The gel was either westernblotted for Mcm10 using Mcm10 (N) antibody or stained with SimplyBlue SafeStain (Invitrogen). Bands at the size of Mcm10 were cut from both Mcm10 and IgG lanes, samples were reduced with dithiothreitol, alkylated with iodoacetamide and in-gel digested with trypsin. The extracted peptide solutions were analysed using nano LC-MS/MS on an LTQ Orbitrap Velos (ThermoFisher, San Jose, CA). The top five hits by peptide number for proteins identified only in Mcm10 IP and not in control IgG IP are presented.

A

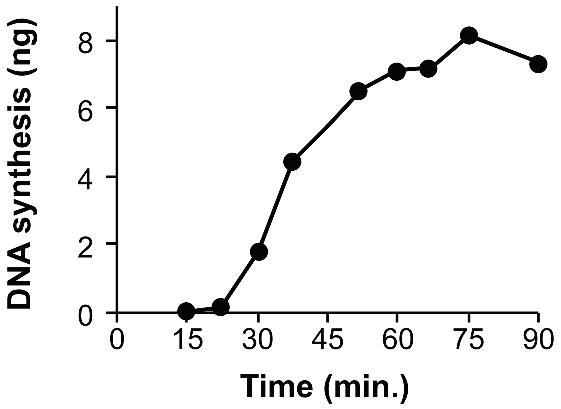

B

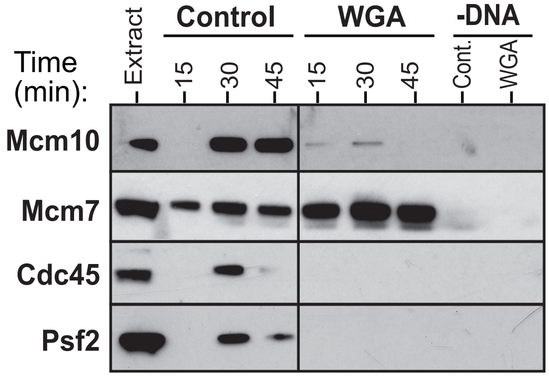

Supplementary Figure S2. Replication in xenopus egg extracts and chromatin binding characteristics of Mcm10.

A, Interphase extract was supplemented with demembranated sperm nuclei and [α-32P]dATP. Total DNA synthesis was determined by stopping the reaction at indicated times. **B**, Interphase egg extracts were supplemented with demembranated sperm nuclei and were optionally supplemented with 2mg/ml wheat germ agglutinin (WGA). At the indicated times, chromatin was isolated and immunoblotted for Mcm10, and CMG components (Mcm7, Cdc45 or Psf2).

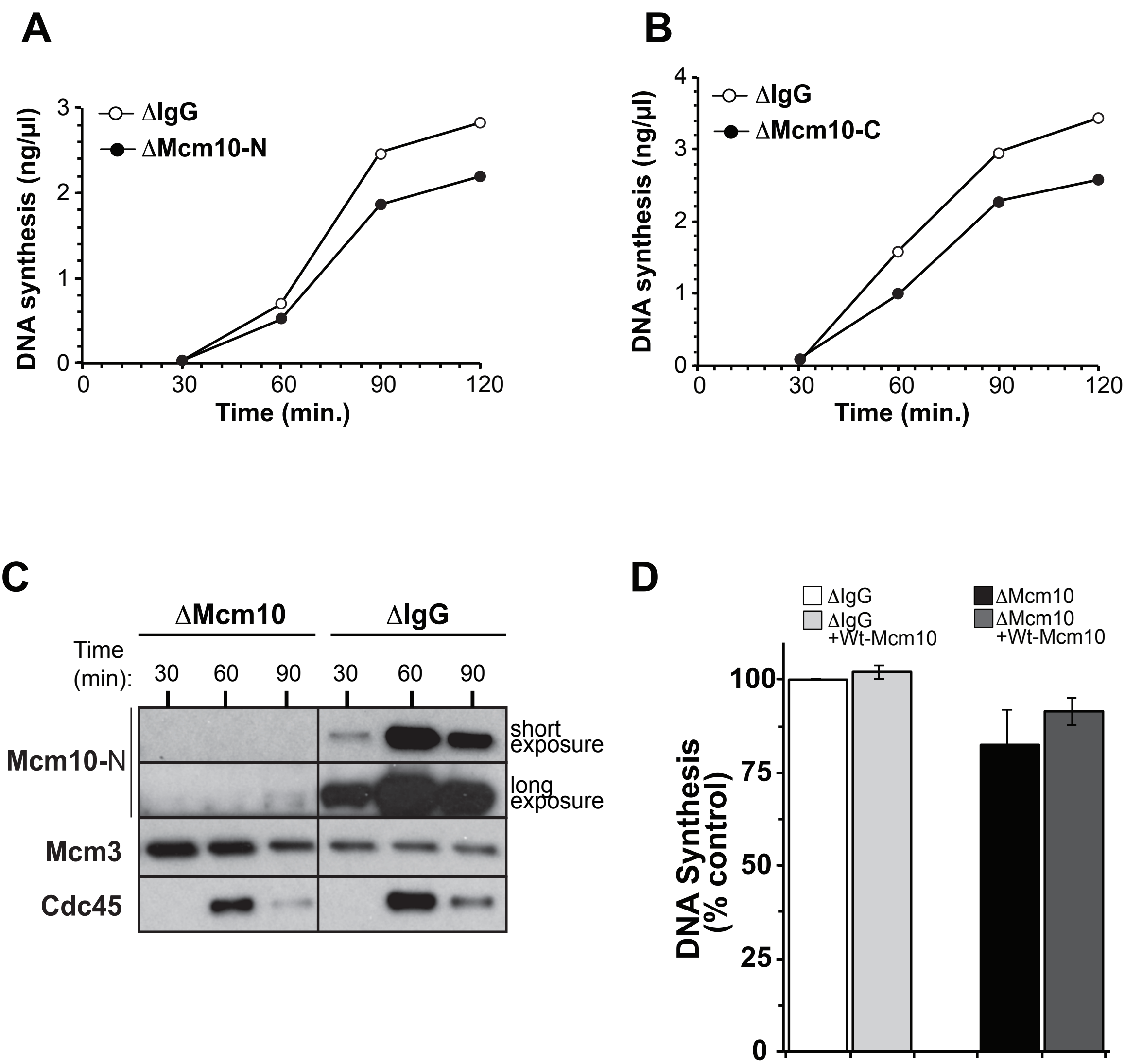

Supplementary Figure S3. Characterisation of Mcm10 immunodepletion in Xenopus egg extracts.

A-B, Egg extract was immunodepleted with either nonimmune IgG or Mcm10 antibodies raised against N-terminus half of the protein (Mcm10-N, A) or raised against C-terminus half of the protein (Mcm10-C, A). Extracts were supplemented with demembranated sperm nuclei and [α-^{32}P]dATP. Total DNA synthesis was determined by stopping the reaction at indicated times. C, Control (nonimmune IgG) and Mcm10 depleted extract were supplemented with demembranated sperm nuclei. After incubation for the indicated times, chromatin was isolated and immunoblotted for Mcm10, Mcm3, and Cdc45. D, Control and Mcm10 depleted extracts were supplemented with demembranated sperm nuclei, [α-32P]dATP and optionally supplemented with wt-Mcm10; total DNA synthesis was determined at 90 min. Mean incorporation of [α-32P]dATP at 90 and S.E.M. of 4 independent experiments is shown.

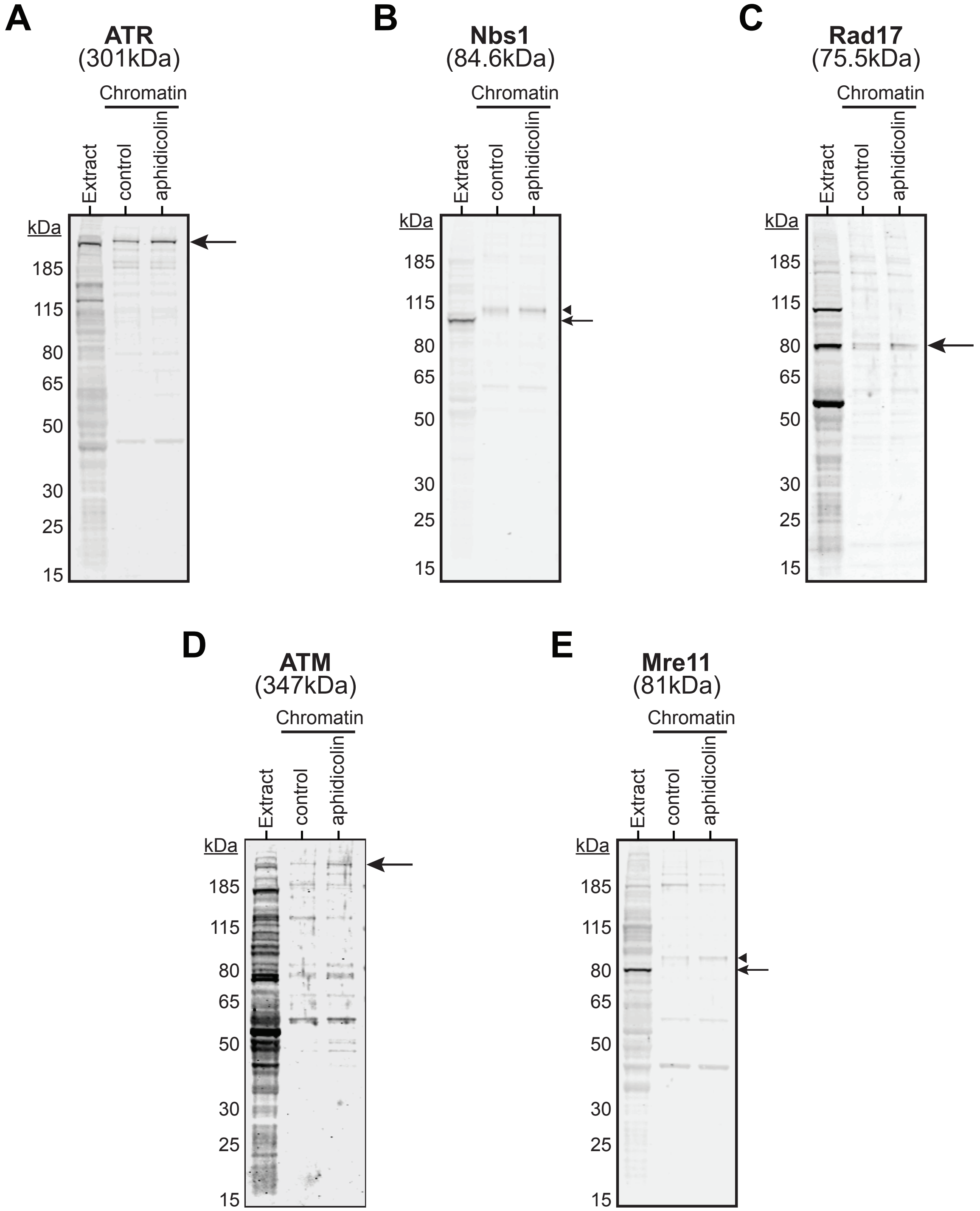

Supplementary Figure S4. Characterisation of antibodies in *Xenopus*.
A-E, 0.5 µl *Xenopus* egg extract, and chromatin isolated after 40 mins from control extract or extract supplemented with 40 µM aphidicolin were separated by SDS-PAGE and immunoblotted with antibodies against *Xenopus* ATR (A), Nbs1 (B), Rad17 (C), ATM (D) and Mre11 (E). Predicted molecular weights of proteins are listed in parenthesis. Migration of protein band on chromatin, where different from extract, is marked with an arrowhead.
